# Racemic 1,2,3,4,7,8,9,10-octa­fluoro-6*H*,12*H*-5,11-methano­dibenzo[*b,f*][1,5]diazo­cine: an octa­fluorinated analogue of Tröger’s base

**DOI:** 10.1107/S1600536808002602

**Published:** 2008-01-30

**Authors:** Christophe M. L. Vande Velde, Delphine Didier, Frank Blockhuys, Sergey Sergeyev

**Affiliations:** a Laboratoire de Chimie des Polymères, Université Libre de Bruxelles, CP 206/1 Boulevard du Triomphe, 1050 Bruxelles, Belgium; b Department of Chemistry, University of Antwerp, Universiteitsplein 1, 2610 Wilrijk, Belgium

## Abstract

The title compound, C_15_H_6_F_8_N_2_, possesses a non-crystal­lographic twofold axis. The dihedral angle between the two benzene rings is 98.4 (2)°. The crystal structure involves intermolecular C—H⋯F hydrogen bonds.

## Related literature

For recent reviews on the chemistry of Tröger’s base (Tröger, 1887 ▶; Spielman, 1935 ▶), see: Valík *et al.* (2005 ▶) and Dolensky *et al.* (2007 ▶). For related literature on the chirality of Tröger’s base, see: Prelog & Wieland (1944 ▶); for mol­ecular clefts, see: Wilcox *et al.* (1987 ▶) and Artacho *et al.* (2006 ▶) and references cited therein; for (poly)halo-substituted Tröger’s base analogues, see: Jensen & Wärnmark (2001 ▶), Sergeyev & Diederich (2004 ▶), Hansson *et al.* (2003 ▶), Li *et al.* (2005 ▶) and Faroughi *et al.* (2006 ▶). For related literature, see: Zabrodsky *et al.* (1993 ▶).
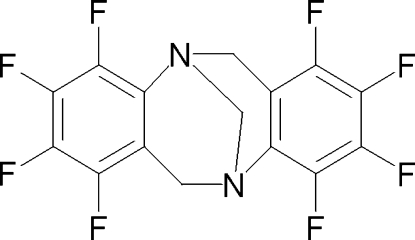


## Experimental

### 

#### Crystal data

C_15_H_6_F_8_N_2_
*M* *_r_* = 366.22
Monoclinic, 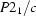

*a* = 8.075 (3) Å
*b* = 10.469 (2) Å
*c* = 17.628 (6) Å
β = 117.15 (2)°
*V* = 1326.0 (8) Å^3^
*Z* = 4
Mo *K*α radiation
μ = 0.19 mm^−1^
*T* = 290 (1) K
0.3 × 0.2 × 0.2 mm

#### Data collection

Enraf–Nonius MACH3 diffractometer
Absorption correction: none
5028 measured reflections
2412 independent reflections
1353 reflections with *I* > 2σ(*I*)
*R*_int_ = 0.078
3 standard reflections every 73 reflections intensity decay: 4%

#### Refinement

*R*[*F* ^2^ > 2σ(*F* ^2^)] = 0.044
*wR*(*F* ^2^) = 0.128
*S* = 1.03
2412 reflections
251 parameters
All H-atom parameters refined
Δρ_max_ = 0.20 e Å^−3^
Δρ_min_ = −0.25 e Å^−3^

### 

Data collection: *CAD-4 EXPRESS* (Enraf–Nonius, 1994 ▶); cell refinement: *CAD-4 EXPRESS*; data reduction: *XCAD4* (Harms & Wocadlo, 1995 ▶); program(s) used to solve structure: *SHELXS97* (Sheldrick, 2008 ▶); program(s) used to refine structure: *SHELXL97* (Sheldrick, 2008 ▶); molecular graphics: *ORTEP-3 for Windows* (Farrugia, 1997 ▶) and *Mercury* (Macrae *et al.*, 2006 ▶); software used to prepare material for publication: *WinGX* (Farrugia, 1999 ▶) and *PLATON* (Spek, 2003 ▶).

## Supplementary Material

Crystal structure: contains datablocks global, I. DOI: 10.1107/S1600536808002602/pk2080sup1.cif

Structure factors: contains datablocks I. DOI: 10.1107/S1600536808002602/pk2080Isup2.hkl

Additional supplementary materials:  crystallographic information; 3D view; checkCIF report

## Figures and Tables

**Table 1:** C—H ⋯ F contacts (Å, °)

C—H⋯F	C—H	H⋯F	C⋯F	C—H⋯F
C13—H132⋯F10^i^	0.93 (3)	2.41 (3)	3.257 (4)	151 (3)
C13—H131⋯F1^ii^	0.98 (4)	2.46 (3)	3.287 (5)	143 (2)
C12—H122⋯F7^iii^	0.98 (3)	2.52 (3)	3.336 (4)	141 (3)

Symmetry codes: (i) 
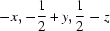
, (ii) 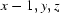, (iii) 
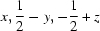
.

**Fig. 1.**
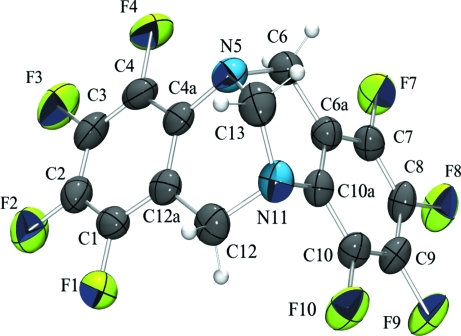
View of the structure with 50% probability displacement ellipsoids. H atoms are numbered according to their attached C-atoms.

**Fig. 2.**
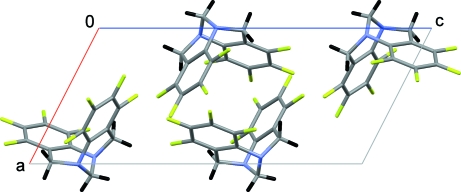
View of the unit cell down the b axis.

## supplementary crystallographic information

### Comment

1,2,3,4,7,8,9,10-Octafluoro-6*H*,12*H*-5,11-methanodibenzo[*b,f*][1,5]diazocine is a fluorinated derivative of Tröger's base (Tröger, 1887; Spielman, 1935), a polycyclic diamine which is chiral due to severely hindered inversion at the bridgehead N-atoms (Prelog & Wieland, 1944). For recent reviews on its chemistry, see Valík *et al.* (2005) and Dolensky *et al.* (2007). Recently, a considerable interest has developed in relatively unfunctionalized receptors with concave aromatic surfaces (termed molecular clefts or tweezers). Wilcox *et al.* (1987) pioneered the incorporation of the Tröger's base framework in chiral molecular clefts by fusing the methanodiazocine core of Tröger's base with two bicyclic aromatic building blocks. Later, molecular clefts comprising two or three fused methanodiazocine cores have been reported (Artacho *et al.*, 2006, and references cited therein). Our interest in the title compound was raised due to the prospect of using highly fluorinated aromatic systems in the design of molecular clefts, thus providing a possibility to explore different supramolecular interactions.

Synthesis of halo-derivatives of Tröger's base was pioneered by Wärnmark (Jensen & Wärnmark, 2001). Later, a number of fluoro-, chloro-, bromo-, and iodo-derivatives of Tröger's base, with the halogen atoms in different positions on the aromatic rings were reported (Sergeyev & Diederich, 2004 and Hansson *et al.*, 2003). However, they typically contain only one halogen atom on each aromatic ring of the methanodiazocine skeleton. Exceptions are the recently reported 2,4,8,10-tetrafluoro- (Li *et al.*, 2005) and tetrabromo-analogs (Faroughi *et al.*, 2006) of Tröger's base. However, polyhalo-analogs of Tröger's base such as the octafluoro analog presented here are unprecedented. To the best of our knowledge, no X-ray crystal structure of a Tröger's base analog with fluorine in the aromatic ring has been reported.

The racemic octafluoro analog of Tröger's base crystallizes in the centrosymmetric space group *P2_1_/c* with one molecule in the asymmetric unit. The molecule has a non-crystallographic twofold symmetry axis through the bridging carbon C13. The CSM (Continuous Symmetry Measure) is 0.0183 (Zabrodsky *et al.*, 1993). Bond lengths and angles are within expectations. TLS analysis returns quasi-isotropic values for the librational amplitudes, and the values for the resulting corrections of the bond lengths are all below the 2σ level. The dihedral angle between the two benzene rings is 98.4 (2)°, which lies within the normal range for analogs of Tröger's base (Dolensky *et al.*, 2007). Cohesion in the structure appears to be mainly provided by aromatic π-π interactions between the fluorinated benzene rings, leading to pairwise ordering of enantiomers around the centers of inversion, with an interplanar distance of under 4 Å. The most clear examples of this in the structure are *Cg*(2)···*Cg*(2)^i^ (^i^=-*x*,1 - *y*,1 - *z*), 3.713 (2) Å, 3.476 (3)Å perp., with a slippage of 1.305 (3) Å, and *Cg*(1) ··· *Cg*(1)^ii^ (^ii^=1 - *x*,-*y*,1 - *z*), 4.805 (2) Å, 3.538 (3)Å perp., with a slippage of 3.251 (3) Å. *Cg*(*x*) indicates the centroid of benzene ring *x*, perp. indicates the perpendicular distance between the ring planes. Due to the lack of suitable hydrogen bond donors, the N-atoms display no close contacts whatsoever. There are a number of F-π contacts in the structure, *e.g.* F(3)···*Cg*(2)^ii^ 3.672 (3) Å, C3—F3···*Cg*(2)^ii^ 161.7 (2)°. Also, H—F contacts occur that are substantially shorter than the van der Waals radii, but these are not usually understood as hydrogen bonds. They are given in Table 1.

### Experimental

2,3,4,5-Tetrafluoroaniline (330 mg, 2 mmol) and paraformaldehyde (120 mg, 4 mmol) were added under vigorous stirring to CF_3_COOH (4 ml) at -15°C. The resulting mixture was allowed to reach room temperature and stirred for 14 days, then slowly added to a stirred mixture of ice and 30% aqueous NH_3_ (7 ml). Extraction with CH_2_Cl_2_ (2 *x* 20 ml), drying of the organic layer over MgSO_4_, and removal of the solvent *in vacuo* gave a crude product which was purified by flash chromatography (SiO_2_/CH_2_Cl_2_). Yield of the title compound: 135 mg (37%). Crystals suitable for X-ray diffraction were grown by slow evaporation from CHCl_3 _solution.

^1^H NMR (300 MHz, CDCl_3_, 25°C): d = 4.20–4.30 (m, 4H, H61, H62, H121, H122), 4.50 (d, J = 17.5 Hz, 2H, H131, H132); ^13^C NMR (75 MHz, CDCl_3_, 25°C): d = 50.1, 66.5, 111.9 (ddt, J =17.7, 3.4, 1.7 Hz), 130.8 (dddd, J = 10.6, 5.2, 3.7, 1.9 Hz), 137.3 (dddd, J = 252.0, 16.5, 13.0, 3.1 Hz), 140.2 (dddd, J = 251.3, 14.9, 13.0, 5.0 Hz), 141.7 (dddd, J = 248.2, 11.2, 4.1, 1.3 Hz), 144.0 (ddt, J = 245.2, 10.9, 3.7 Hz). HR—EI—MS: m/*z*: calcd. for C_15_H_6_F_8_N_2_ ([*M*]^+^): 366.0403; found: 366.0405.

### Refinement

Hydrogen atoms were located in the Fourier difference map and refined freely. (C—H 0.93 (3)–1.03 (3) Å)

### Crystal data

**Table:**

C_15_H_6_F_8_N_2_	*D*_x_ = 1.834 Mg m^−^^3^
*M**_r_* = 366.22	Melting point: 192(1) K
Monoclinic, *P*2_1_/*c*	Mo *K*α radiation λ = 0.71073 Å
Hall symbol: -P 2ybc	Cell parameters from 25 reflections
*a* = 8.075 (3) Å	θ = 5–12º
*b* = 10.469 (2) Å	µ = 0.19 mm^−^^1^
*c* = 17.628 (6) Å	*T* = 290 (1) K
β = 117.15 (2)º	Cell measurement pressure: 101(1) kPa
*V* = 1326.0 (8) Å^3^	Rhomb, colourless
*Z* = 4	0.3 × 0.2 × 0.2 mm
*F*_000_ = 728	

### Data collection

**Table:**

Enraf–Nonius MACH3 diffractometer	*R*_int_ = 0.078
Radiation source: sealed tube	θ_max_ = 25.3º
Monochromator: pyrolytic graphite	θ_min_ = 2.3º
*T* = 290(1) K	*h* = 0→9
*P* = 101(1) kPa	*k* = −12→12
profiled ω/2θ scans	*l* = −21→18
Absorption correction: none	3 standard reflections
5028 measured reflections	every 73 reflections
2412 independent reflections	intensity decay: 4%
1353 reflections with *I* > 2σ(*I*)	

### Refinement

**Table:**

Refinement on *F*^2^	Hydrogen site location: difference Fourier map
Least-squares matrix: full	All H-atom parameters refined
*R*[*F*^2^ > 2σ(*F*^2^)] = 0.044	*w* = 1/[σ^2^(*F*_o_^2^) + (0.06*P*)^2^] where *P* = (*F*_o_^2^ + 2*F*_c_^2^)/3
*wR*(*F*^2^) = 0.128	(Δ/σ)_max_ < 0.001
*S* = 1.03	Δρ_max_ = 0.20 e Å^−^^3^
2412 reflections	Δρ_min_ = −0.25 e Å^−^^3^
251 parameters	Extinction correction: SHELXL97 (Sheldrick, 2008), Fc^*^=kFc[1+0.001xFc^2^λ^3^/sin(2θ)]^-1/4^
Primary atom site location: structure-invariant direct methods	Extinction coefficient: 0.008 (2)
Secondary atom site location: difference Fourier map	

### Special details

**Table:**

Geometry. All e.s.d.'s (except the e.s.d. in the dihedral angle between two l.s. planes) are estimated using the full covariance matrix. The cell e.s.d.'s are taken into account individually in the estimation of e.s.d.'s in distances, angles and torsion angles; correlations between e.s.d.'s in cell parameters are only used when they are defined by crystal symmetry. An approximate (isotropic) treatment of cell e.s.d.'s is used for estimating e.s.d.'s involving l.s. planes.

### Fractional atomic coordinates and isotropic or equivalent isotropic displacement parameters (Å^2^)

**Table:**

	*x*	*y*	*z*	*U*_iso_*/*U*_eq_	
H131	−0.193 (4)	0.216 (3)	0.8025 (17)	0.051 (8)*	
H121	0.305 (4)	0.121 (3)	0.810 (2)	0.069 (9)*	
H122	0.136 (5)	0.176 (3)	0.726 (2)	0.077 (10)*	
H62	−0.105 (5)	0.310 (3)	0.9326 (17)	0.057 (9)*	
H132	−0.111 (4)	0.269 (3)	0.741 (2)	0.060 (9)*	
H61	0.092 (4)	0.374 (3)	0.9788 (19)	0.053 (9)*	
F7	0.1474 (3)	0.21118 (18)	1.10195 (11)	0.0715 (6)	
F10	0.2539 (3)	−0.08189 (16)	0.87533 (11)	0.0699 (6)	
F1	0.5029 (3)	0.29145 (19)	0.78363 (12)	0.0679 (6)	
F9	0.3839 (3)	−0.15905 (16)	1.03755 (11)	0.0729 (6)	
N11	0.0509 (3)	0.1395 (2)	0.81513 (13)	0.0506 (6)	
F8	0.3308 (3)	−0.01382 (19)	1.15148 (10)	0.0730 (6)	
C12A	0.2581 (4)	0.3151 (3)	0.81963 (16)	0.0467 (7)	
F4	0.1600 (3)	0.58222 (17)	0.92368 (14)	0.0845 (7)	
F3	0.4753 (3)	0.66558 (18)	0.92211 (15)	0.0919 (7)	
C10A	0.1288 (4)	0.1067 (3)	0.90262 (15)	0.0417 (6)	
C9	0.2933 (4)	−0.0464 (3)	1.01318 (17)	0.0489 (7)	
F2	0.6451 (3)	0.5220 (2)	0.85025 (14)	0.0852 (6)	
C7	0.1727 (4)	0.1397 (3)	1.04471 (16)	0.0471 (7)	
N5	−0.0035 (3)	0.3514 (2)	0.85287 (15)	0.0570 (7)	
C4A	0.1674 (4)	0.3903 (3)	0.85490 (17)	0.0488 (7)	
C1	0.4148 (4)	0.3626 (3)	0.81791 (18)	0.0510 (8)	
C8	0.2668 (4)	0.0260 (3)	1.07054 (16)	0.0491 (7)	
C10	0.2252 (4)	−0.0074 (3)	0.93048 (16)	0.0450 (7)	
C6A	0.1055 (4)	0.1831 (3)	0.96176 (17)	0.0460 (7)	
C13	−0.0834 (5)	0.2423 (4)	0.7960 (2)	0.0611 (9)	
C2	0.4898 (4)	0.4799 (3)	0.8519 (2)	0.0583 (8)	
C4	0.2450 (5)	0.5081 (3)	0.88958 (18)	0.0584 (8)	
C3	0.4040 (5)	0.5512 (3)	0.8886 (2)	0.0616 (9)	
C6	0.0168 (5)	0.3137 (3)	0.9374 (2)	0.0577 (9)	
C12	0.1908 (5)	0.1805 (3)	0.78815 (19)	0.0530 (8)	

### Atomic displacement parameters (Å^2^)

**Table:**

	*U*^11^	*U*^22^	*U*^33^	*U*^12^	*U*^13^	*U*^23^
F7	0.1095 (15)	0.0648 (11)	0.0538 (10)	0.0031 (11)	0.0490 (11)	−0.0071 (9)
F10	0.1081 (15)	0.0474 (10)	0.0537 (10)	0.0049 (10)	0.0364 (10)	−0.0103 (8)
F1	0.0709 (12)	0.0683 (12)	0.0764 (12)	0.0122 (10)	0.0440 (10)	0.0101 (10)
F9	0.0981 (15)	0.0433 (10)	0.0660 (12)	0.0137 (10)	0.0276 (10)	0.0114 (9)
N11	0.0534 (14)	0.0571 (16)	0.0335 (12)	0.0002 (13)	0.0130 (10)	0.0035 (11)
F8	0.1033 (15)	0.0666 (12)	0.0379 (9)	0.0000 (11)	0.0224 (9)	0.0098 (9)
C12A	0.0484 (17)	0.0506 (16)	0.0352 (14)	0.0092 (14)	0.0139 (12)	0.0113 (13)
F4	0.1074 (17)	0.0539 (11)	0.1061 (16)	0.0224 (11)	0.0607 (14)	−0.0004 (11)
F3	0.1082 (17)	0.0477 (11)	0.1105 (17)	−0.0089 (12)	0.0419 (14)	−0.0055 (12)
C10A	0.0435 (15)	0.0430 (14)	0.0348 (14)	−0.0044 (13)	0.0144 (12)	0.0002 (12)
C9	0.0557 (17)	0.0351 (15)	0.0481 (16)	0.0008 (14)	0.0170 (14)	0.0039 (13)
F2	0.0694 (13)	0.0731 (13)	0.1142 (16)	−0.0077 (11)	0.0429 (12)	0.0129 (12)
C7	0.0589 (18)	0.0477 (17)	0.0397 (15)	−0.0058 (15)	0.0268 (13)	−0.0068 (13)
N5	0.0536 (15)	0.0572 (15)	0.0577 (15)	0.0156 (14)	0.0233 (12)	0.0161 (13)
C4A	0.0512 (17)	0.0468 (17)	0.0455 (16)	0.0137 (15)	0.0195 (14)	0.0172 (14)
C1	0.0523 (17)	0.0547 (19)	0.0478 (16)	0.0145 (16)	0.0244 (14)	0.0120 (14)
C8	0.0609 (18)	0.0467 (16)	0.0316 (14)	−0.0078 (15)	0.0140 (13)	0.0058 (13)
C10	0.0570 (17)	0.0389 (15)	0.0376 (14)	−0.0047 (14)	0.0201 (13)	−0.0074 (13)
C6A	0.0489 (17)	0.0457 (16)	0.0443 (15)	0.0000 (13)	0.0220 (13)	0.0014 (13)
C13	0.0468 (19)	0.078 (2)	0.0480 (19)	0.0021 (17)	0.0123 (15)	0.0164 (18)
C2	0.0519 (19)	0.0541 (18)	0.067 (2)	0.0070 (16)	0.0250 (16)	0.0183 (16)
C4	0.072 (2)	0.0476 (18)	0.0587 (19)	0.0222 (17)	0.0330 (17)	0.0108 (16)
C3	0.067 (2)	0.0402 (17)	0.067 (2)	0.0048 (16)	0.0217 (17)	0.0091 (15)
C6	0.058 (2)	0.058 (2)	0.064 (2)	0.0141 (17)	0.0335 (18)	0.0116 (17)
C12	0.063 (2)	0.0587 (19)	0.0358 (15)	0.0005 (16)	0.0215 (15)	0.0011 (14)

### Geometric parameters (Å, °)

**Table:**

F7—C7	1.345 (3)	F2—C2	1.342 (3)
F10—C10	1.347 (3)	C7—C8	1.373 (4)
F1—C1	1.348 (3)	C7—C6A	1.384 (4)
F9—C9	1.350 (3)	N5—C4A	1.423 (4)
N11—C10A	1.417 (3)	N5—C13	1.461 (4)
N11—C13	1.454 (4)	N5—C6	1.476 (4)
N11—C12	1.476 (4)	C4A—C4	1.393 (5)
F8—C8	1.343 (3)	C1—C2	1.380 (5)
C12A—C1	1.372 (4)	C6A—C6	1.513 (4)
C12A—C4A	1.400 (4)	C13—H131	0.98 (3)
C12A—C12	1.520 (4)	C13—H132	0.93 (3)
F4—C4	1.346 (3)	C2—C3	1.365 (5)
F3—C3	1.343 (4)	C4—C3	1.368 (5)
C10A—C10	1.387 (4)	C6—H62	0.95 (3)
C10A—C6A	1.393 (4)	C6—H61	0.95 (3)
C9—C8	1.357 (4)	C12—H121	1.03 (3)
C9—C10	1.365 (4)	C12—H122	0.98 (3)
			
C10A—N11—C13	110.2 (2)	C7—C6A—C10A	118.7 (3)
C10A—N11—C12	113.3 (2)	C7—C6A—C6	120.2 (3)
C13—N11—C12	108.1 (3)	C10A—C6A—C6	121.0 (3)
C1—C12A—C4A	118.7 (3)	N11—C13—N5	111.7 (2)
C1—C12A—C12	120.5 (3)	N11—C13—H131	112.5 (18)
C4A—C12A—C12	120.7 (3)	N5—C13—H131	106.2 (17)
C10—C10A—C6A	118.3 (2)	N11—C13—H132	105.1 (19)
C10—C10A—N11	119.5 (2)	N5—C13—H132	107 (2)
C6A—C10A—N11	122.1 (3)	H131—C13—H132	114 (3)
F9—C9—C8	119.9 (3)	F2—C2—C3	120.8 (3)
F9—C9—C10	119.8 (3)	F2—C2—C1	120.8 (3)
C8—C9—C10	120.3 (3)	C3—C2—C1	118.4 (3)
F7—C7—C8	119.0 (2)	F4—C4—C3	119.3 (3)
F7—C7—C6A	119.3 (3)	F4—C4—C4A	119.2 (3)
C8—C7—C6A	121.7 (3)	C3—C4—C4A	121.6 (3)
C4A—N5—C13	111.1 (3)	F3—C3—C2	119.2 (3)
C4A—N5—C6	113.0 (2)	F3—C3—C4	120.3 (3)
C13—N5—C6	107.1 (3)	C2—C3—C4	120.5 (3)
C4—C4A—C12A	118.1 (3)	N5—C6—C6A	110.4 (3)
C4—C4A—N5	120.1 (3)	N5—C6—H62	106.7 (17)
C12A—C4A—N5	121.8 (3)	C6A—C6—H62	109 (2)
F1—C1—C12A	119.3 (3)	N5—C6—H61	109.5 (17)
F1—C1—C2	118.0 (3)	C6A—C6—H61	109.4 (18)
C12A—C1—C2	122.7 (3)	H62—C6—H61	112 (2)
F8—C8—C9	120.2 (3)	N11—C12—C12A	110.5 (3)
F8—C8—C7	120.5 (3)	N11—C12—H121	113.2 (18)
C9—C8—C7	119.3 (2)	C12A—C12—H121	107.9 (19)
F10—C10—C9	118.6 (3)	N11—C12—H122	109 (2)
F10—C10—C10A	119.8 (2)	C12A—C12—H122	111 (2)
C9—C10—C10A	121.6 (3)	H121—C12—H122	105 (3)
			
C13—N11—C10A—C10	164.7 (3)	F7—C7—C6A—C6	4.4 (4)
C12—N11—C10A—C10	−74.1 (3)	C8—C7—C6A—C6	−174.9 (3)
C13—N11—C10A—C6A	−12.6 (4)	C10—C10A—C6A—C7	−2.5 (4)
C12—N11—C10A—C6A	108.7 (3)	N11—C10A—C6A—C7	174.8 (2)
C1—C12A—C4A—C4	−2.4 (4)	C10—C10A—C6A—C6	174.6 (3)
C12—C12A—C4A—C4	174.4 (3)	N11—C10A—C6A—C6	−8.1 (4)
C1—C12A—C4A—N5	175.1 (2)	C10A—N11—C13—N5	53.2 (3)
C12—C12A—C4A—N5	−8.2 (4)	C12—N11—C13—N5	−71.2 (3)
C13—N5—C4A—C4	165.8 (3)	C4A—N5—C13—N11	51.4 (3)
C6—N5—C4A—C4	−73.8 (3)	C6—N5—C13—N11	−72.5 (3)
C13—N5—C4A—C12A	−11.6 (3)	F1—C1—C2—F2	0.4 (4)
C6—N5—C4A—C12A	108.8 (3)	C12A—C1—C2—F2	178.8 (3)
C4A—C12A—C1—F1	−179.6 (2)	F1—C1—C2—C3	−178.4 (3)
C12—C12A—C1—F1	3.7 (4)	C12A—C1—C2—C3	0.0 (4)
C4A—C12A—C1—C2	2.0 (4)	C12A—C4A—C4—F4	179.9 (2)
C12—C12A—C1—C2	−174.7 (3)	N5—C4A—C4—F4	2.4 (4)
F9—C9—C8—F8	0.1 (4)	C12A—C4A—C4—C3	0.9 (4)
C10—C9—C8—F8	178.7 (3)	N5—C4A—C4—C3	−176.6 (3)
F9—C9—C8—C7	−179.3 (3)	F2—C2—C3—F3	1.1 (5)
C10—C9—C8—C7	−0.8 (4)	C1—C2—C3—F3	179.9 (3)
F7—C7—C8—F8	0.7 (4)	F2—C2—C3—C4	179.6 (3)
C6A—C7—C8—F8	179.9 (3)	C1—C2—C3—C4	−1.6 (5)
F7—C7—C8—C9	−179.8 (3)	F4—C4—C3—F3	0.7 (4)
C6A—C7—C8—C9	−0.6 (4)	C4A—C4—C3—F3	179.7 (3)
F9—C9—C10—F10	−1.3 (4)	F4—C4—C3—C2	−177.8 (3)
C8—C9—C10—F10	−179.9 (3)	C4A—C4—C3—C2	1.2 (5)
F9—C9—C10—C10A	179.0 (3)	C4A—N5—C6—C6A	−75.7 (4)
C8—C9—C10—C10A	0.5 (4)	C13—N5—C6—C6A	47.0 (3)
C6A—C10A—C10—F10	−178.4 (2)	C7—C6A—C6—N5	166.7 (3)
N11—C10A—C10—F10	4.2 (4)	C10A—C6A—C6—N5	−10.4 (4)
C6A—C10A—C10—C9	1.2 (4)	C10A—N11—C12—C12A	−75.3 (3)
N11—C10A—C10—C9	−176.2 (3)	C13—N11—C12—C12A	47.2 (3)
F7—C7—C6A—C10A	−178.5 (3)	C1—C12A—C12—N11	166.2 (2)
C8—C7—C6A—C10A	2.3 (4)	C4A—C12A—C12—N11	−10.5 (4)

### Table 1 C—H ··· F contacts (Å, °) (i) -x, -1/2+y, 1/2-z (ii) x-1, y, z (iii) x, 1/2-y, -1/2+z

**Table:**

C—H···F	C—H	H···F	C···F	C—H···F
C13—H132···F10^i^	0.93 (3)	2.41 (3)	3.257 (4)	151 (3)
C13—H131···F1^ii^	0.98 (4)	2.46 (3)	3.287 (5)	143 (2)
C12—H122···F7^iii^	0.98 (3)	2.52 (3)	3.336 (4)	141 (3)
